# Intra-tumoural extra-cellular pH: a useful parameter of response to chemotherapy in syngeneic tumour lines

**DOI:** 10.1038/sj.bjc.6605022

**Published:** 2009-04-14

**Authors:** D Lindner, D Raghavan

**Affiliations:** 1 Cleveland Clinic, Taussig Cancer Institute, 9500 Euclid Avenue, R35, Cleveland, OH 44195, USA

**Keywords:** pH, intra-tumoural, extra-cellular, response, chemotherapy, syngeneic tumour

## Abstract

Reliable surrogate markers of response to anticancer therapy remain a desirable tool for preclinical modelling and clinical practice in oncology. Clinical evaluation is relatively unreliable when attempting to assess rapidly and prospectively the outcome of treatment. Fluxes in released or circulating tumour marker levels are a useful but inconsistent marker of cytotoxic response. Serial measurement of circulating tumour cells appears to have some utility as a surrogate marker, but assay systems are expensive, and many cancers are not associated with the presence of circulating tumour cells. Because tissue breakdown is associated with release of nucleic acids and other cellular products, we reasoned that serial measurement of intra-tumoural pH may correlate with the extent of tumour lysis, and thus with outcomes of cytotoxic chemotherapy. Doxorubicin-sensitive and doxorubicin-resistant sublines of P388 murine monocytic leukaemia in C57BL/6 mice were treated with increasing concentrations of doxorubicin. Tumours were serially measured by conventional bi-dimensional methods and pH was sampled using a bevelled tip electrode. Mean and median pH changes were statistically different in responsive and resistant tumours, and amplitude of change correlated with long-term responses to doxorubicin. Serial sampling of pH in tumour masses may provide a useful surrogate of long-term response to chemotherapy.

The clinical assessment of cytotoxic response to a chemotherapy agent or regimen is a relatively standardised process, involving the documentation of initial extent of disease and the use of serial measurement by clinical or radiological techniques (Husband *et al*, 2004). This process usually takes place during a period of several weeks or months of chemotherapy, and the first assessment is usually carried out after the delivery of 2–3 cycles of treatment, usually correlating with a time lapse of 2–3 months. The limitations of clinical assessment of tumour response have been detailed extensively (Warr *et al*, 1984; Therasse *et al*, 2005), but this paradigm has been the best available technique, although patients destined not to respond may suffer unnecessary toxicities of therapy until the emerging therapeutic failure is recognised.

More recently, serial assessment of tumour antigens (Hussain *et al*, 2006) and measurement of circulating tumour cells (Cristofanilli *et al*, 2004; de Bono *et al*, 2008) have been tested as surrogate markers of response to treatment. Although in several collaborative studies, we have shown that each approach has utility (Cristofanilli *et al*, 2004; Hussain *et al*, 2006; de Bono *et al*, 2008), important limitations have been identified: many tumours do not produce tumour-associated antigens, or there will be discordance between fluctuations in serial levels and response to treatment (Cristofanilli *et al*, 2004). Similarly, some tumours are not associated with the elaboration of tumour cells in the circulation, in which case the latter assay has only a limited function (Budd *et al*, 2006).

It is well known that cell death or necrosis is associated with breakdown of cancer tissues, with the release of nucleic acids and other intra-cellular components (Tredan *et al*, 2007). In fact, tumours *per se* are often associated with an acidic extra-cellular environment (Wike-Hooley *et al*, 1985). In highly responsive tumours, such as lymphomas and the paediatric malignancies, cytotoxic treatment of large volume cancer can be associated with tumour lysis syndrome, with rapid and catastrophic release of large amounts of intra-cellular material into the blood stream, culminating in acidosis and acute renal failure (Tiu *et al*, 2007).

We have reasoned that, in less extreme situations, effective chemotherapy may still be associated with less dramatic release of breakdown products, with associated acidosis, and that the serial measurement of extra-cellular pH within individual tumour masses may show that the extent of response correlates with the extent of pH reduction in extra-cellular fluid. We propose that this could provide a simple and reproducible early surrogate of tumour response that might be available as a guide to treatment earlier than conventional means, and that might even be a more accurate long-term predictor of outcome. Thus we hypothesise that a responsive tumour will undergo early tumour necrosis in response to chemotherapy and that this will lead to local release of nucleic acids, in turn correlating with a measurable fall in pH within the tumour. We further hypothesise that the extent of pH change will correlate with duration of response. We have thus developed a preclinical model, contrasting the clones of cytotoxic-responsive and cytotoxic-resistant cell lines, to address this hypothesis.

## Materials and methods

### Cell lines

P388 murine monocytic leukaemia cells (ATCC, Manassas, VA, USA) were maintained in RPMI-1640 (Mediatech Inc., Herndon, VA, USA) and 5% FBS (HyClone, Logan, UT, USA). The parental doxorubicin (adriamycin, ADR)-sensitive and the ADR-resistant sublines of B16-BL6 mouse melanoma (selected by exposure to increasing concentrations of ADR *in vitro*) were a gift from Dr Ram Ganapathi (Cleveland Clinic, Cleveland, OH, USA). B16-BL6 cells were maintained in DMEM (Mediatech Inc.) and 5% FBS (HyClone) without ADR. The resistant subline was adapted to grow in the presence of 0.25 *μ*g ml^−1^ ADR in monolayer culture. Resistance was confirmed in antiproliferative assays (Figure 4). The tumourigenicity in C57BL/6 mice of the ADR-resistant subline was similar to parental sensitive cells following subcutaneous (s.c.) implantation of 1 million tumour cells. All cell lines were confirmed mycoplasma free by PCR.

### Reagents

Cyclophosphamide monohydrate (Sigma-Aldrich, St Louis, MO, USA) and ADR (doxorubicin HCl; Sigma-Aldrich) were used in these studies. Cyclophosphamide (Cy) was prepared in DMSO (15 mg ml^−1^) and given as a single 100 mg kg^−1^ intraperitoneal (i.p.) dose on day 9 during treatment of mice bearing P388 leukaemia. Doxorubicin (2 mg kg^−1^ i.p.) was given daily for 6 days, starting on day 6 after B16-BL6 tumour cell inoculation. Doses were based on previous experience and reflected maximum tolerated mouse doses that were associated with tumour response with tolerable toxicity (Ganapathi *et al*, 1988). Control mice received phosphate-buffered saline (PBS), consisting of monobasic potassium phosphate (1.54 mM), dibasic sodium phosphate (2.71 mM), and sodium chloride (155 mM), pH 7.2.

### Antiproliferative assays

Cells were treated with doxorubicin during growth in RPMI-1640 (Mediatech Inc.) and 5% FBS (HyClone). Doxorubicin was added 6 h after plating cells to allow cells to adhere to the wells before drug exposure. Growth was monitored using a colorimetric assay (Skehan *et al*, 1990). Each treatment group contained eight replicates. Cells were fixed and stained with sulphorhodamine B after 4 days. Bound dye was eluted from cells and absorbance (*A*_exp_) was measured at 570 nm. One plate was fixed 8 h after plating to determine the absorbance representing starting cell number (*A*_ini_). Absorbance with this plate and that obtained with untreated cells at the end of the growth period (*A*_fin_) were taken as 0 and 100% growth, respectively. Thus, 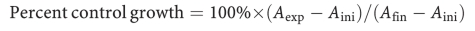 Expressed as a percent of untreated controls, a decrease in cell number (relative to starting cell number) is a negative number on the *y*-axis.

### Measurement of pH *in vivo*

DBA/2 mice (*n*=24 each group) were inoculated in the flank with 0.1 × 10^6^ P388 murine monocytic leukaemia cells. Tumours were allowed to grow for 9 days and reached approximately 8–10 mm diameter. On day 9, control mice received PBS; experimental mice received a single dose of Cy 100 mg kg^−1^ i.p. After 2 days, pH measurements were obtained.

C57Bl6 mice (*n*=16 each group) were inoculated subcutaneously in the flank with 0.1 million ADR-sensitive or ADR-resistant B16-BL6 cells. On day 6 after tumour inoculation, control mice started receiving PBS and experimental mice received doxorubicin, 2 mg kg^−1^ i.p. daily. Treatment was continued for a total of 6 days, followed by tumour pH measurements.

#### Single pH measurements

An Orion 98–63 micro-pH electrode (Thermo Electron Corp., Beverly, MA, USA), a 16-gauge bevelled tip electrode (1.6 mm OD) coupled to an Orion 310 PerpHecT pH meter (Thermo Electron Corp.), was used to measure intra-tumoural pH. The electrode was autocalibrated with pH 4.00 and 7.00 buffers (Corning, Woburn, MA, USA) before each series of measurements. Mice were anaesthetised with ketamine/xylazine/acepromazine (50/5/1 mg kg^−1^ i.p.) before pH measurement. The bevelled pH probe was inserted transcutaneously into the tumours. Measurements were taken in the central region of the tumours as well as in the tumour-free s.c. region of the posterior cervical midline. Each experiment was performed three times with similar results. Student's *t*-test (two-tailed homoscedastic) was utilised to determine *P*-values and statistical significance between groups.

#### Kinetics of intra-tumoural pH

C57Bl6 mice (*n*=6 each group) were inoculated subcutaneously in the mid-dorsal lumbar region with 0.1 million ADR-sensitive or ADR-resistant B16-BL6 cells. On day 4 after tumour inoculation, 2-mm-diameter tubes with ion-selective polyvinyl chloride membranes covering the distal end (Kwik-Tip-H; World Precision Instruments, Sarasota, FL, USA) were introduced through an incision in the dorsal thoracic region, tunnelled through the dorsal s.c. space, and the distal tip placed in the centre of the tumour. The incision was closed and the tube was fixed in place with 5-0 Vicryl suture; thus, tubes remained *in situ* for the duration of the study. On day 6, control mice started receiving PBS and experimental mice received doxorubicin, 2 mg kg^−1^ i.p. daily, continued for a total of 6 days. Following administration of general anaesthesia as above, tumour pH was assessed on day 6 (5 min pre- and 5 min post-doxorubicin), and on days 7, 10, 13, and 16 by introduction of the calibrated Kwik-Tip-H electrode into the implanted tube containing 1 M citric acid and 0.01 M NaCl (pH 5.6). The 2 mm polyvinyl chloride-coated reference electrode (SDR2; World Precision Instruments) was introduced through a separate 2 mm incision in the flank. It was recognised that, if this preclinical approach were successful, a modified system of measurement would be required for routine clinical application, and design of such a system is in progress.

#### Animal welfare

All murine studies were conducted under compliance with the Cleveland Clinic Institutional Animal Care and Use Committee, under Animal Welfare Assurance A3047-01.

## Results

**Figure 1:**
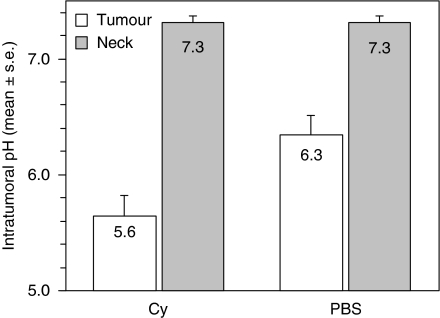
Measurement of extra-cellular pH in P388 murine monocytic leukaemia tumours and normal tissues in DBA/2 mice. Extra-cellular pH measurements of established (11-day-old) subcutaneous tumours and the subcutaneous tissues of the posterior neck were obtained using a micro-electrode. Mice had received PBS or cyclophosphamide (Cy) 2 days previously (*n*=24 each group).

**Figure 2:**
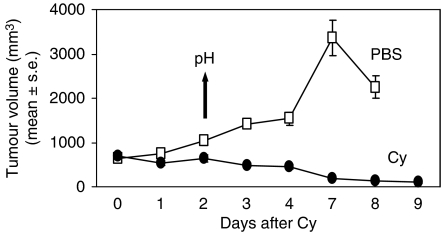
P388 tumour volume in DBA/2 mice. Mice bearing established s.c. P388 tumours received 100 mg kg^−1^ Cy on day 0. Intra-tumoural pH was assessed on day 2 (indicated by arrow), as above.

**Figure 3:**
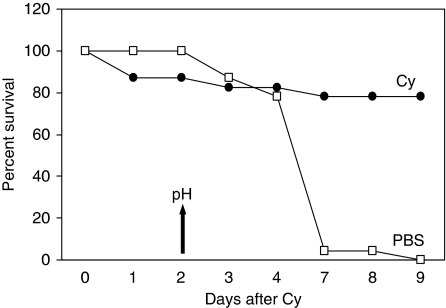
Survival of mice bearing P388 tumours. Mice from Figure 2 were monitored for survival following single-dose of Cy or vehicle.

P388 murine leukaemia is exquisitely sensitive to the cytotoxic effects of Cy. To determine whether tumour regression was associated with changes in tumour pH, we treated mice bearing established s.c. syngeneic tumours with a single dose of Cy or PBS. Tumours in both treatment groups were acidotic at baseline, compared to normal s.c. tissue (Figure 1). The lowest tumour mean pH was observed following Cy treatment compared to PBS (5.6±0.2 and 6.3±0.2, respectively, *P*=0.001). There was no difference in the pH measured in the s.c. tissue in the cervical region in Cy- and PBS-treated mice (7.3 for both, *P*=0.97). Tumours grew progressively in the PBS group, whereas tumours underwent regression in the Cy group (Figure 2). Cyclophosphamide treatment resulted in 80% complete remission and long-term survival (90 days), whereas there were no survivors in the PBS-treated group (Figure 3). Hence, long-term survival was predicted by induction of acidosis within tumours.

**Figure 4:**
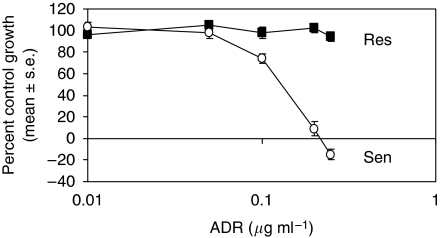
Antiproliferative effects of doxorubicin (ADR) against B16-BL6 murine melanoma. Doxorubicin sensitive (Sen) and resistant (Res) sublines of B16-BL6 were treated with doxorubicin *in vitro* and growth was assessed after 4 days. Negative values on the *y*-axis indicate death of initially plated cells (*n*=8 each dose).

To determine if this phenomenon was cell type specific, we selected a murine melanoma model consisting of drug-resistant and drug-sensitive sublines. We have previously used this model extensively for other unrelated studies of cytotoxicity and clonality of response (Ganapathi *et al*, 1988). We recognised that the drug responsive sublines of a melanoma xenograft do not necessarily translate to responsiveness in the clinical setting, but simply used this line as a comparative model of cytotoxic response and resistance for the purposes of illustrating this general phenomenon. Antiproliferative assays were conducted to determine the *in vitro* ADR_IC50_ values for both B16-BL6 sublines. Adriamycin-resistant cells were not growth inhibited by 0.01–0.25 *μ*g ml^−1^ ADR, whereas the ADR_IC50_ for ADR-sensitive cells was approximately 0.15 *μ*g ml^−1^ (Figure 4). Hence, the ADR-resistant cells proved to be resistant *in vitro*.

**Figure 5:**
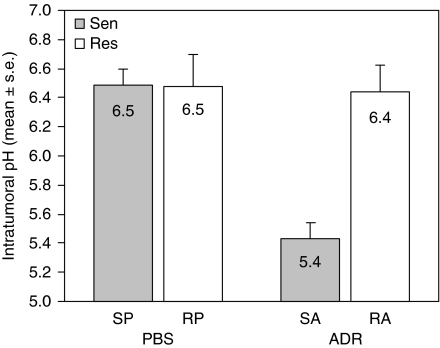
Measurement of intra-tumoural pH in B16-BL6 tumours. Extra-cellular pH measurements of sensitive and resistant B16-BL6 tumours were obtained following doxorubicin (ADR, 2 mg kg^−1^ × 6 days) or PBS treatment. SP (sensitive, PBS), RP (resistant, PBS), SA (sensitive, ADR), RA (resistant, ADR) designations indicate cell subline and treatment (*n*=16 mice each group).

**Figure 6:**
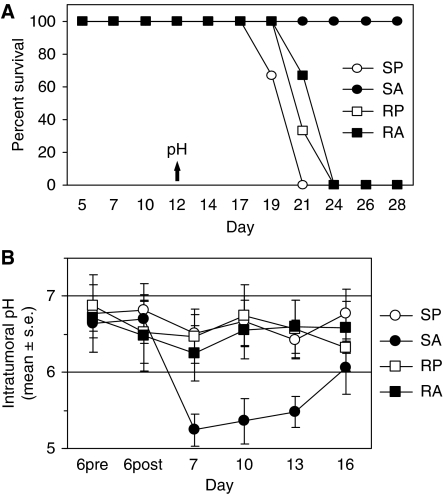
Survival of C57Bl6 mice bearing B16-BL6 tumours and kinetics of pH response. (**A**) Mice implanted with tumours as in Figure 5 were monitored for survival following treatment with ADR or PBS (*n*=16). Intra-tumoural pH was measured at a single time point (day 12, indicated by arrow). Cell subline and treatment designations as in Figure 5. (**B**) A second series of C57Bl6 mice (*n*=6 per group) bearing B16-BL6 tumours with indwelling intra-tumoural guide tubes received ADR or PBS treatment (days 6–12) and underwent repetitive pH measurement on days 6 (pre- and post-treatment), 7, 10, 13, and 16.

The B16-BL6 sublines were established as s.c. tumours in C57Bl6 mice, followed by a 6-day course of either PBS or ADR. Following ADR treatment, ADR-sensitive tumours displayed a lower intra-tumoural pH compared to resistant tumours (5.4±0.1 and 6.4±0.2, respectively, *P*=3.5 × 10^−7^) (Figure 5). There was no significant difference in cervical s.c. pH (representing normal, non-malignant tissues at a close but not contiguous site) between ADR-sensitive or ADR-resistant tumour-bearing mice, whether they were treated with ADR or PBS (data not shown). By 12 days following the last treatment, all mice bearing ADR-resistant tumours had died (or were terminated because of excessive tumour size), as had mice bearing ADR-sensitive tumours treated with PBS (Figure 6A). However, mice with ADR-sensitive tumours that had received ADR displayed 100% survival. Kinetic studies indicated that the maximal drop in pH occurred 24 h post-treatment (*P*<0.02, SA *vs* other three curves, days 7, 10, 13) (Figure 6B). Hence, induction of tumour acidosis again translated into a positive therapeutic response.

## Discussion

In this preclinical study, we have demonstrated clearly that intra-tumoural, extra-cellular pH changes dramatically in response to two different chemotherapy regimens in responsive tumours. In contrast, chemotherapy-resistant tumours are not associated with this change in pH, and control tumour-bearing mice treated with saline show no pH changes. Not surprisingly, long-term survival has correlated directly with the extent of pH change, reflecting the extent of initial tumour cell kill. This model suggests that early intra-tumour measurement of pH change in the clinical setting may predict outcome of chemotherapy, and thus may provide a useful tool to allow earlier prediction in clinical practice.

It is possible that the local changes in acidity are not merely a reflection of cell kill, but may be part of the process. It has been shown that tumour necrosis factor-related apoptosis-inducing ligand (TRAIL) is a potential anticancer agent that induces a greater level of necrosis-like cell death under acidic conditions than at physiological pH (Meurette *et al*, 2007), and this may reflect induction of caspase function (Lee *et al*, 2004; Meurette *et al*, 2005). A range of other proteases are induced at acidic pH, and while one usually thinks of these in association with mechanisms of invasion and metastasis, it is possible that they also contribute to local tumour destruction once a cascade of cell necrosis has been initiated. Our future preclinical studies will focus on the interactions of expression of TRAIL, a series of proteases and the balance of apoptosis and necrosis in response to cytotoxic chemotherapy.

However, at present our data strongly support the concept that measurement of extra-cellular pH changes will allow clinical prediction of response to chemotherapy, and we are initiating a clinical trial to test this hypothesis, using single needle probe measurement of pH at set time points after initial cytotoxic chemotherapy.

The obvious problem with this approach is the innate, potential discomfort for patients, notwithstanding the use of a scaled-down probe size that we are developing. Furthermore, this measurement technology would only be applicable to easily accessed tumour deposits, such as cutaneous metastases, localised breast cancers, and lymph node masses. However, another potential tool that could exploit the same underlying pathophysiology would be the use of non-invasive magnetic resonance spectroscopy to measure similar end points, and this could much more easily be applied to visceral tumour deposits. This also will be the subject of future studies.
